# Clinical manifestations of cuticular drusen in Korean patients

**DOI:** 10.1038/s41598-020-68493-2

**Published:** 2020-07-10

**Authors:** Dong Hoon Shin, Mingui Kong, Gyule Han, Jong Chul Han, Don-Il Ham

**Affiliations:** 1 Hangil Eye Hospital Retina Center, Incheon, South Korea; 2 Department of Ophthalmology, Samsung Medical Center, Sungkyunkwan University School of Medicine, 81 Irwon-ro, Gangnam-gu, Seoul, 06351 South Korea

**Keywords:** Visual system, Retinal diseases, Macular degeneration, Vision disorders

## Abstract

Cuticular drusen show some similarities to and differences from soft drusen in age-related macular degeneration (AMD) and might thus be a unique AMD subtype. Previous studies on cuticular drusen were performed mainly in white ethnic groups, but AMD shows ethnic differences. We investigated clinical manifestations of cuticular drusen in Korean patients to evaluate possible ethnic differences. Clinical records of Korean patients with cuticular drusen were retrospectively reviewed. Fundus distribution pattern, imaging features, and presence of large drusen, drusenoid pigment epithelial detachment (PED), and macular complications, including geographic atrophy (GA), choroidal neovascularization (CNV), and acquired vitelliform lesion (AVL), were assessed via multimodal imaging in 162 eyes with cuticular drusen (n = 81 patients; 67 females; mean age: 66.6 ± 9.1 years). Diffuse distribution was found in 61.7% and peripapillary involvement in 75.3% of eyes. Large drusen, drusenoid PED, GA, CNV, and AVL were observed in 59.3%, 26.5%, 18.5%, 3.7%, and 1.2% of eyes, respectively. The macular complication prevalence was similar between patients ≤ 60 and those > 60 years old. In Korean patients, cuticular drusen were less frequently associated with macular complications than in white patients, and the proportion of macular complications differed significantly, with AVL representing an uncommon complication.

## Introduction

Cuticular drusen are multiple, small, round drusen that appear hyperfluorescent in fluorescein angiography (FA), giving a “stars-in-the-sky” appearance^1,2^. A histopathological study revealed that cuticular drusen are located between the basal lamina of the retinal pigment epithelium (RPE) and the inner collagenous layer of Bruch’s membrane, similar to the soft drusen in age-related macular degeneration (AMD)^3,4^. Imaging studies using spectral domain optical coherence tomography (SD-OCT) have also shown the similarity in location of cuticular drusen and soft drusen^4,5^.

Although cuticular drusen share many features in common with the soft drusen in AMD, there are also several differences between these two types of drusen. Cuticular drusen are more widely scattered on the fundus, with symmetrical distribution patterns in both eyes. In cuticular drusen, the age of onset is younger and genetic associations are stronger than in AMD^6–8^. In addition, smoking, one of the most important environmental factors in AMD, is weakly associated with cuticular drusen^9^. Cuticular drusen can cause vision loss as a result of vitelliform macular detachment, and it could be related to membranoproliferative glomerulonephritis type II and other renal diseases^2,4,10,11^. Thus, cuticular drusen might be a unique subtype of AMD, with particular characteristics.

Previous studies on cuticular drusen were performed mainly in white ethnic groups, and clinical features in other ethnic groups remain largely unknown^4,6,10,12–14^. Yet, there are known ethnic differences in AMD. The prevalence of AMD varies in different ethnic groups, and the prevalence of subtypes of neovascular AMD also varies; for instance, polypoidal choroidal vasculopathy (PCV) is more prevalent in Asians than in white ethnic groups^15–18^. Considering ethnic differences in AMD and proposed genetic associations in cuticular drusen, there might also be ethnic differences in the manifestation of cuticular drusen^18^.

Thus, this study aimed to broaden our understanding of cuticular drusen by examining multimodal imaging features, clinical phenotypes, and prevalence of macular complications in Korean patients. Additionally, we sought clues to the possible existence of ethnic differences in the manifestation of cuticular drusen.

## Results

Of the 210 eyes of 105 patients previously diagnosed with cuticular drusen, 162 eyes of 81 Korean patients were included in this study. Forty-eight eyes were excluded for the following reasons: in 22 eyes, drusen were distributed only in the peripheral retina; in 4 eyes, multimodal imaging data were insufficient; in 2 eyes, there was a history of previous vitreoretinal surgery; 2 eyes had severe cataract, and 18 eyes had accompanying diabetic retinopathy.

Demographic and clinical features are summarized in Table 1. All patients were Koreans, and 67 patients were female (82.7%). Eleven (13.4%) patients had various types of kidney disease, including renal cyst, polycystic kidney disease, renal stone, and acute kidney injury associated with the ingestion of herbs. However, no patients had membranoproliferative glomerulonephritis type II, which was previously reported to be associated with cuticular drusen^11^.

**Table 1:** Clinical and demographic features.

Feature	Data
No. of eyes	162
No. of patients	81
Women	67 (82.7)
Age (years)	66.6 ± 9.1
BCVA (logMAR)	0.15 ± 0.28
Refraction (spherical equivalent)	0.20 ± 1.43
SFCT (µm)	220.1 ± 85.3
**Ocular comorbidities**	
Pseudophakia	34 (20.1)
Epiretinal membrane	7 (4.3)
Glaucoma/glaucoma suspect	8 (4.9)
**Medical history**	
Diabetes	16 (19.8)
Hypertension	40 (49.4)
Thyroid disease	11 (13.6)
Kidney disease	11 (13.6)

Data are total no. (%) or mean ± standard deviation, unless otherwise indicated.

*BCVA *best-corrected visual acuity, *logMAR *logarithm of the minimum angle of resolution, *SFCT *subfoveal choroidal thickness.

### Multimodal imaging

All study subjects underwent color fundus photography (CFP), spectral-domain optical coherence tomography (SD-OCT), and FA. All individual imaging results met the diagnostic criteria. Red-free (RF), near-infrared reflectance (NIR), and fundus autofluorescence (FAF) imaging were performed in 50.6%, 97.5%, and 96.3% of patients, respectively. Ultrawide-field photographic images were acquired in 48.1% of patients. Imaging characteristics are summarized in Table 2. A macular-type distribution pattern was seen in 62 eyes (38.3%) and a diffuse-type in 100 eyes (61.7%).The average age of patients with each type of distribution pattern was 65.8 ± 8.4 and 67.1 ± 9.6 years, respectively, which was not statistically significantly different (*P* = 0.522, Student’s *t* test). Peripapillary involvement and occupation of the total retinal area by cuticular drusen in more than five disc-areas were observed in 122 eyes (75.3%) and in 134 eyes (82.7%), respectively.

**Table 2:** Imaging characteristics of cuticular drusen.

Characteristics	Data
**Distribution pattern of drusen**	
Macula	62 (38.3)
Diffuse	100 (61.7)
**Involved fundus area**	
Peripapillary involvement	122 (75.3)
Occupied retinal area ≥ 5DA	134 (82.7)
**Accompanying macular lesions**	
Large drusen	96 (59.3)
Drusenoid PED	43 (26.5)
Geographic atrophy	30 (18.5)
CNV	6 (3.7)
Vitelliform lesion	2 (1.2)

Data are total no. (%), unless otherwise indicated.

*CNV *choroidal neovascularization, *DA *disc-areas, *PED *pigment epithelial detachment.

**Figure 1:**
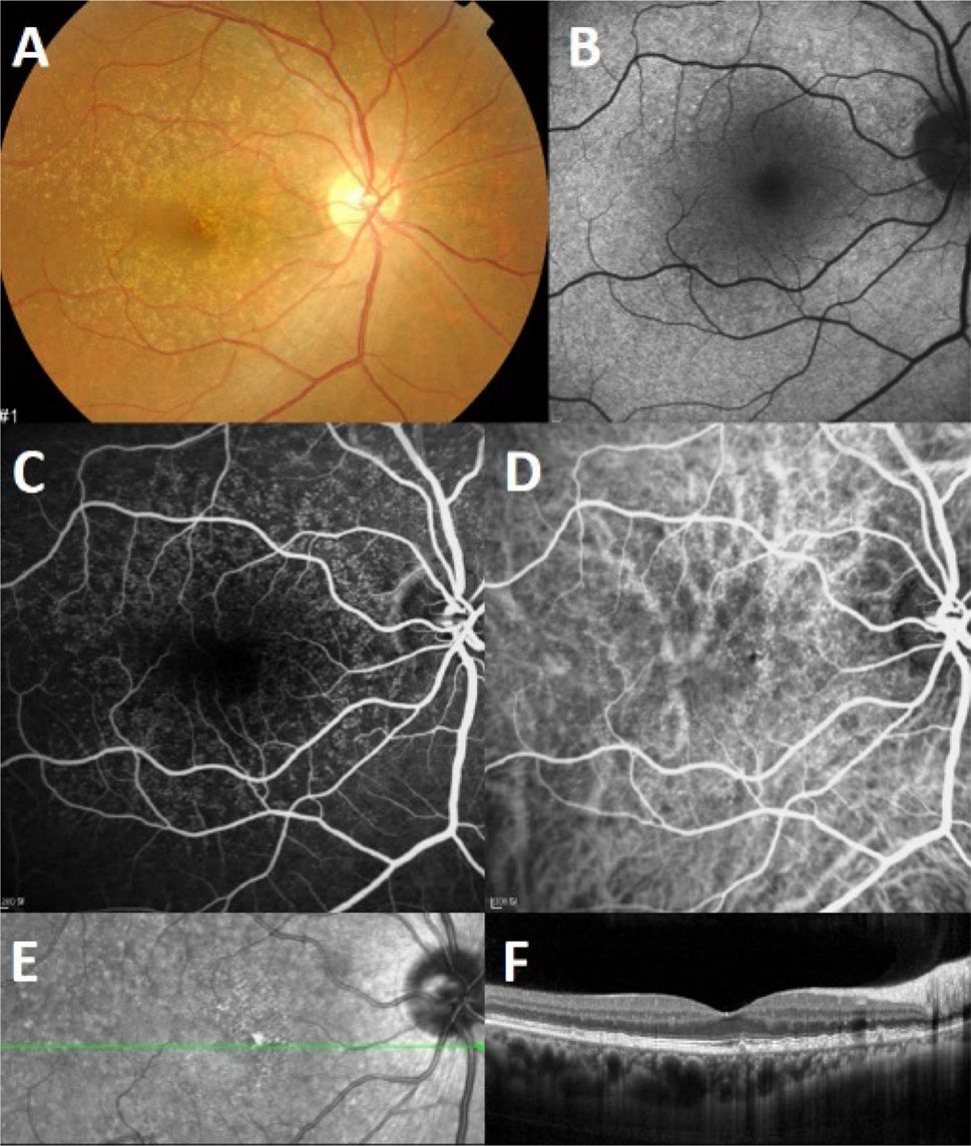
Multimodal imaging of cuticular drusen. CFP (**A**) shows multiple scattered pale or yellow spots. A hypoautofluorescent center with a mild hyperautofluorescent margin was observed on FAF image (**B**). “Stars-in-the-sky” hyperfluorescent lesions are observed in the early phase of FA (**C**) and ICGA (**D**). A combination of a hyporeflective center and hyperreflectivity is seen on the NIR image (**E**). A “sawtooth pattern” of small and confluent sub-RPE deposits is observed in OCT image (**F**). *CFP *color fungus photography, *FA *fluorescein angiography, *FAF *fundus autofluorescence, *ICGA *indocyanine green angiography, *NIR *near-infrared reflectance, *OCT *optical coherence tomography, *RPE *retinal pigment epithelium.

Multimodal imaging features of cuticular drusen in Korean patients were similar to those reported in patients of white ethnicities. Cuticular drusen were seen as hyperchromic dots on RF images in all eyes. NIR imaging of cuticular drusen showed hyporeflective centers with a surrounding hyperreflective margin (63.3%), diffuse hyperreflectivity (19.0%), heterogeneous reflectivity (3.8%), or a combination of these patterns (13.9%). The characteristic FAF finding of cuticular drusen, i.e., central hypoautofluorescence and a rim of hyperautofluorescence, was observed in 65.4% of eyes. The remaining eyes showed nonspecific findings of FAF. The morphological findings of cuticular drusen on SD-OCT were categorized into three patterns: type 1 pattern (37.0%), type 2 pattern (43.2%), and type 3 pattern (19.8%). Indocyanine green angiography (ICGA) showed hyperfluorescence in 60% of eyes (18/30). Figure 1 shows the multimodal imaging characteristics of cuticular drusen.

**Figure 2:**
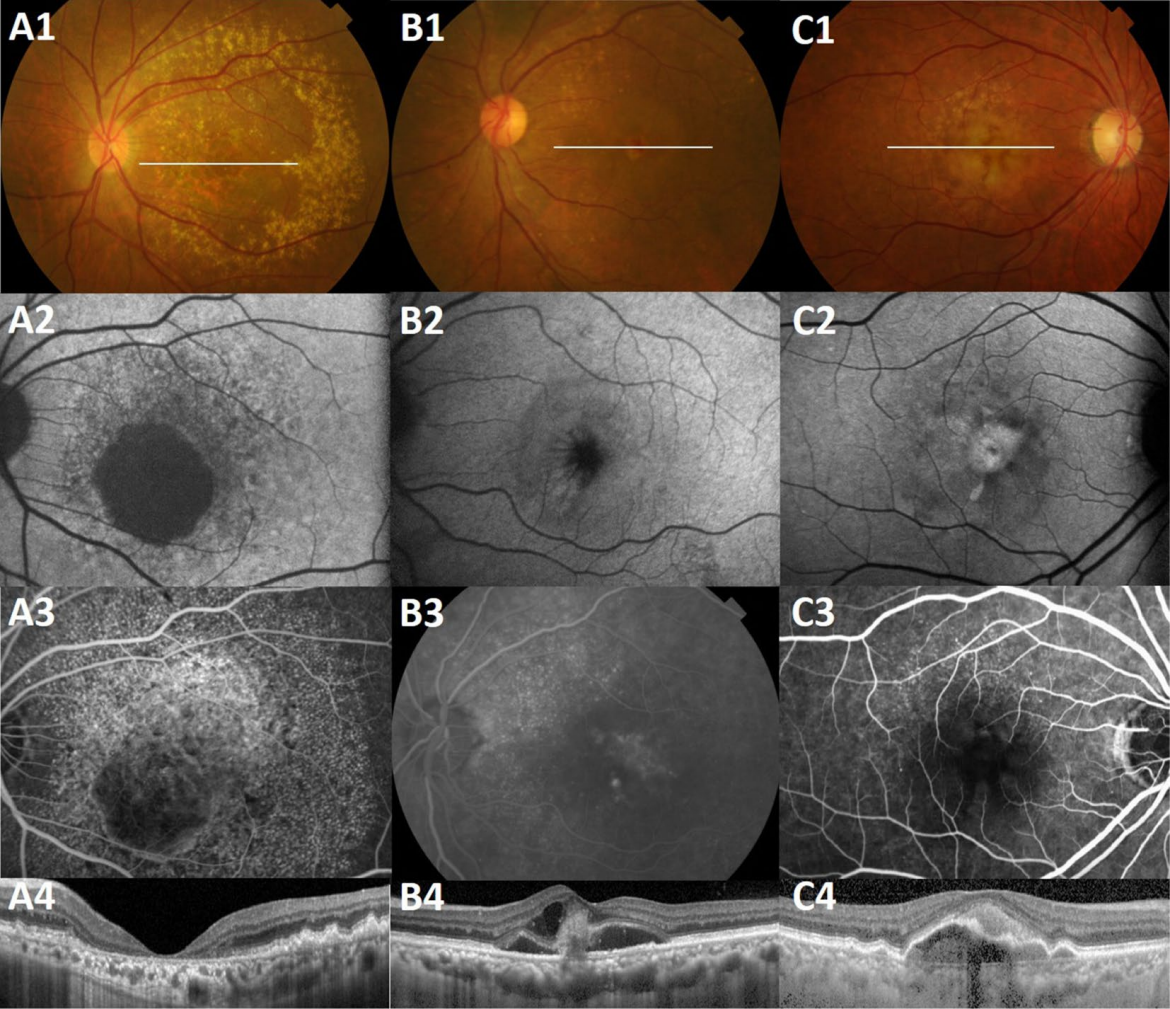
Macular complications of cuticular drusen. (**A**) Geographic atrophy is seen on (**A1**) CFP, (**A2**) FAF, (**A3**) FA, and (**A4**) OCT in a 65-year-old man with cuticular drusen. Geographic atrophy appears in both eyes symmetrically. Large drusen are also visible on CFP and OCT. (**B**) Type 3 choroidal neovascularization is seen on (**B1**) CFP, (**B2**) FAF, (**B3**) FA, and (**B4**) OCT in a 67-year-old woman with cuticular drusen. (**C**) Drusenoid pigment epithelial detachment and acquired vitelliform lesion appear as (**C1**) round yellow lesions on CFP, (**C2**) intense hyperautofluorescence on FAF, (**C3**) hypofluorescence in the early phase of FA, and a (**C4**) subretinal location of vitelliform material with pigment epithelial detachment is seen on OCT, in a 74-year-old man. The position of the OCT line scans is shown in the CFP as a white transverse line. *CFP *color fundus photograph, *FA *fluorescein angiography, *FAF *fundus autofluorescence, *OCT *optical coherence tomography.

In some eyes, large drusen (59.3%, Fig. 2A) were scattered among cuticular drusen, and drusenoid pigment epithelial detachment (PED, 26.5%, Fig. 2C) accompanied the cuticular drusen. Some eyes were complicated by geographic atrophy (GA, 18.5%, Fig. 2A), choroidal neovascularization (CNV, 3.7%, Fig. 2B), and acquired vitelliform lesion (AVL, 1.2%, Fig. 2C). Six eyes with CNV were classified as follows: 1 eye (0.6%) with type 1, 2 eyes (1.2%) with type 2, 2 eyes (1.2%) with a mixture of type 1 and type 2, and 1 eye (0.6%) with type 3. There was no PCV. The previously reported prevalence rates of GA, CNV, and AVL in cuticular drusen in the white ethnic groups and Japanese patients are summarized in Table 3. Statistical analysis of the relationship between age and the prevalence macular complications revealed no statistically significant differences between patients ≤ 60 years and those > 60 years old (Table 4).

**Table 3:** Comparison of recent cuticular drusen studies.

Author(s), year	No. of patients	Sex (female:male)	Mean Age (years)	Region	Imaging tools for diagnosis of CD	GA (%)	Neovascular AMD (%)	AVL (%)
Cohen et al., 1994^14^	19	13:6	61.4	France	CFP, FA^a^	0	31.6^c^	100^d,e^
Barbazetto et al., 2007^6^	28	17:11	65.9	United States	CFP, AF, FA^b^	3.6^d^	3.6^d^	100^d,e^
Boon et al., 2008^10^	30	20:10	53.3	Netherlands	CFP, NIR, OCT, AF, FA^a^	33.3^d^	16.7^d^	26.7^d^
Boon et al., 2013^12^	198	–	–	Netherlands	CFP, RF, NIR, OCT, AF, FA^a^, ICGA	–	56^d^	–
Balaratnasingam et al., 2018^4^	120	72:48	57.9	United States, Australia, France	CFP, RF, NIR, OCT, AF, FA^b^, ICGA	25.0^c^	12.5^c^	24.2^c^
Sakurada et al., 2019^13^	12	8:4	60.8	Japan	CFP, NIR, OCT, AF, FA^a^, ICGA	8.3^c^	4.2^c^	0
Sakurada et al., 2019^19^,^f^	38	35:3	59.9	United States	CFP, FAF, OCT, FA^a^	14.5^f^	2.6^f^	–
Current study, 2018	81	67:14	66.6	Korea	CFP, RF, NIR, OCT, AF, FA^a^, ICGA	18.5^c^	3.7^c^	1.2^c^

– unknown, *AF *fundus autofluorescence, *AMD *age-related macular degeneration, *AVL *acquired vitelliform lesion, *CD *cuticular drusen, *CFP *color fundus photography, *FA *fluorescein angiography, *GA *geographic atrophy, *ICGA *indocyanine green angiography, *NIR *near-infrared, *OCT *optical coherence tomography, *RF *red-free imaging.

^a^FA was performed in all patients.

^b^FA was performed in selected patients.

^c^% of eyes.

^d^% of patients.

^e^Patients who had both vitelliform or pseudovitelliform detachment and cuticular drusen were included.

^f^Baseline cross-sectional data before exclusion.

**Table 4:** Age-related variations in macular complications of cuticular drusen.

	Entire cohort (n = 162)	Age ≤ 60 years (n = 36)	Age > 60 years (n = 126)	P-value^a^
GA	30 (18.5)	5 (13.9)	25 (19.8)	0.417^b^
CNV	6 (3.7)	1 (2.8)	5 (4.0)	1.000^c^
AVL	2 (1.2)	0 (0)	2 (1.6)	1.000^c^

Data are number (%) or mean ± standard deviation, unless otherwise indicated.

*AVL *acquired vitelliform lesion, *CNV *choroidal neovascularization, *GA *geographic atrophy, *PED *pigment epithelial detachment.

^a^Comparison between eyes in patients ≤ 60 years and those > 60 years of age.

^b^Chi-square test.

^c^Fisher’s exact test.

Peripheral reticular pigmentary degeneration was observed in 68 eyes of 34 patients (42.0%), and all patients had a symmetric degeneration pattern in both eyes. Peripheral reticular pigmentary degeneration was present in 6 of 31 patients (19.4%) with the macular-type and 28 of 50 patients (56.0%) with the diffuse-type distribution pattern. The diffuse-type distribution of cuticular drusen was more strongly associated with the presence of peripheral reticular pigmentary degeneration than the macular-type (*P* = 0.001; Chi-square test).

## Discussion

There have been only a limited number of reports on cuticular drusen in Asian ethnic groups, and one recent study reported on about 12 Japanese patients^13^. In the present study, we report on a sizeable number of Korean patients (81) with cuticular drusen.

We report several similarities in demographic characteristics and clinical findings associated with cuticular drusen between Koreans and white ethnic groups. There was a trend toward female sex being a risk factor for cuticular drusen in white ethnic groups, with a female preponderance of proportion of 60.0–92.1%^4,6,9,10,12–14,19^. A similar female preponderance was observed in our Korean patients (82.7%) as well as in the recent Japanese study (66.6%)^13^. There were also similarities in the fundus distribution pattern between the white ethnic groups and Korean patients. For the diffuse-type distribution pattern, previous studies on white ethnic groups reported a prevalence of 67.1–81%, while the prevalence in Korean patients in this study was 61.7%^4,12^.

Most multimodal imaging features of cuticular drusen in Korean patients revealed no significant differences from those reported in patients of white ethnicities. For example, types 1, 2, and 3 OCT patterns were seen in 37.0%, 43.2%, and 19.8% of Korean patients, and in 33%, 49%, and 18% of patients of white ethnicities^4^. The FAF and ICGA findings were also similar between these ethnic groups.

Peripheral cuticular drusen were reported to be associated with senile reticular pigmentary degeneration in white ethnic groups^10,12^. In Korean patients, peripheral reticular pigmentary degeneration was present in 42% of patients, and the diffuse distribution-type was more strongly associated with peripheral reticular pigmentary degeneration than the macular-type (56% vs 19.4%). However, we could not compare the finding in Korean patients to those in white ethnic groups, because previous studies in white ethnic groups used different diagnostic criteria and classification methods.

However, the current data also revealed several noticeable differences in the clinical manifestation of cuticular drusen between Koreans and white ethnic groups (Table 3). According to the present study, Korean patients have GA far more frequently than CNV (18.5% vs 3.7%). One recent, small study reported similar findings in a white ethnic group; however, most previous studies on patients of white ethnicity reported smaller differences between the prevalence of GA and CNV. These studies reported that CNV could develop in 3.6–56% of patients in white ethnic groups^4,6,10,12,14^. In Korean patients, CNV was observed in only 6 eyes (3.7%) of 5 patients (6.2%); thus, the frequency of CNV in Koreans appeared to be relatively lower than the frequencies reported in white ethnic groups.

A similar ethnic difference was reported for exudative AMD. A previous study reported that Asian Americans had a lower risk of exudative AMD than in white Americans^15^. Interestingly, none of the eyes with CNV had PCV lesions, which is the preponderant neovascularization subtype in Asians^20^. A single hospital-based study reported that the prevalence of PCV in Korean exudative AMD patients was 24.6%^21^.

Around one-quarter (24.2–26.7%) of eyes with cuticular drusen in white ethnic groups reportedly develop vitelliform lesions^4,10^. However, only 1.2% of eyes among our Korean patients had vitelliform lesions. A recent Japanese study found no AVL in 12 patients, supporting the finding of a low frequency of AVL in Korean patients^13^. Thus, it appears that Korean patients might have AVL less frequently than white ethnic groups; however, prospective, longitudinal studies are needed to verify these findings.

In our analysis of the relationship between aging and the prevalence of macular complications, we found that, unlike in patients of white ethnicities, there were no statistically significant differences in macular complications between patients ≤ 60 years and those > 60 years old^4^. We found a similar result when we changed the age cut-off to 65 or 70 years (data not shown).

Recently, Sakurada et al. reported that drusenoid PED was the most frequent AMD-associated macular pathology in eyes of Japanese patients (33.3%)^13^. In Korean patients, drusenoid PED was seen in 26.5% of eyes. The frequency of drusenoid PED in white ethnic groups has not been reported. Further studies are therefore needed to compare drusenoid PED between Asians and white ethnic groups.

Previous studies of cuticular drusen have used various diagnostic criteria (Table 3). Cohen et al. used the simple criteria based on the characteristic FA finding, while Van de Ven et al. used more complex criteria based on size, location, symmetry, and number of drusen, in addition to the characteristic FA finding^9,14^. In a recent large observational cohort study by Balaratnasingam et al., cuticular drusen were diagnosed by a combination of multiple imaging modalities, and FA was not mandatory^4^. In the present study, strict diagnostic criteria were used with multimodal imaging. CFP, OCT, and FA were used in the diagnosis of all patients, and NIR and FAF images were also assessed in more than 96% of patients.

A previous study suggested that cuticular drusen might initially develop in the peripheral fundus during the early phase of the disease^8,10,12^. Of 210 eyes in 105 Korean patients previously diagnosed with cuticular drusen, 22 eyes had drusen distributed only in the peripheral retina. However, they were excluded from analysis in this study, because we consider that there is currently insufficient evidence that these lesions are the same as the cuticular drusen observed at the posterior fundus. Further studies including longitudinal studies are needed to understand the nature of peripheral drusen showing hyperfluorescence in FA.

The current study had several limitations. The study was retrospective and cross-sectional. The results from Korean patients were indirectly compared to those from white ethnic groups reported in previous studies, which used slightly different diagnostic criteria and assessment methods. Eyes having only peripheral lesions were excluded from analysis in this study; further studies are needed to verify the nature of these peripheral lesions. The number of patients ≤ 60 years old was markedly smaller than the number of patients > 60 years old, which may have affected statistical analyses.

In conclusion, Korean patients with cuticular drusen showed features similar to those previously reported in white ethnic groups with regard to female preponderance, distribution pattern, and most multimodal imaging characteristics. However, the cuticular drusen were less frequently associated with macular complications in Korean patients than in white ethnic groups, and AVL was found rarely. Thus, we thought that this study provides the preliminary evidences for future prospective studies to investigate ethnic differences in cuticular drusen.

## Methods

### Participants

Clinical records of patients diagnosed with cuticular drusen at the retina clinic of Samsung Medical Center, Seoul, Korea, between January 2010 and July 2018 were retrospectively reviewed. Patients who had other retinal disorders that interfered with the assessment of angiography images, including diabetic retinopathy, hypertensive retinopathy, and retinal vein occlusion, were excluded. Patients who had ocular conditions that might affect the evaluation of cuticular drusen, such as severe cataract, previous trauma history, and previous vitreoretinal surgery history, were also excluded.

Demographic information, including age, sex, ocular and systemic comorbidities, and medical history were obtained for each patient. All patients underwent ophthalmic examinations including measurement of best-corrected visual acuity (BCVA), refractive error by manifest refraction, slit-lamp biomicroscopy, and dilated fundus examination. Patients underwent multimodal imaging, including CFP (TRC 50 IX, Topcon, Tokyo, Japan), RF, NIR, FAF imaging, and SD-OCT (Spectralis HRA + OCT, Heidelberg Engineering, Heidelberg, Germany). FA, ICGA (Spectralis HRA + OCT or Optos 200Tx, Optos PLC, Dunfermline, United Kingdom), and ultrawide-field photography (Optos 200Tx) were also performed.

### Definition of cuticular drusen and AMD

The current study used strict diagnostic criteria for cuticular drusen, mainly based on the definition originally proposed by Gass and the criteria proposed by Van de Ven et al. and by Balaratnasingam et al.^1,4,9^. In brief, cuticular drusen were defined as multiple, yellow or pale, small, round lesions observed in fundus examination and CFP, showing a symmetric distributed pattern between bilateral eyes. There should be at least 50 scattered, uniformly-sized, small (25–75 μm) hyperfluorescent drusen with a typical “stars-in-the-sky” appearance on FA images in each eye^4,9^. The lesions had to be located beneath the RPE, with RPE elevation on OCT images^4,24^. NIR and FAF imaging features were used to assist the diagnosis, although neither of these imaging tests were considered as mandatory for diagnosis^4,23^.

Fundus topographic distribution patterns of cuticular drusen were classified as either the macular- or the diffuse-type, based on the results of CFP and FA^4^. Briefly, a macular distribution-type was defined as drusen distributed only within the major vascular arcades, whereas the diffuse-type was defined as drusen involving the macula, but also extending beyond the vascular arcades. Involvement of the peripapillary region and the total retinal area occupied by cuticular drusen, exceeding 5 disc-areas, were also analyzed^4^.

Multimodal imaging data were assessed for RF findings, FAF findings, NIR patterns, morphologic features on OCT b-scan, and ICGA findings. NIR patterns were categorized as hyporeflective centers with a surrounding hyperreflective margin, diffuse hyperreflectivity, heterogeneous reflectivity, or a combination of these patterns^4^. Morphological features on OCT b-scans were divided into a type 1 pattern, showing shallow elevation of the RPE-basal laminar band with difficult to discern internal drusen contents; a type 2 pattern, showing a sawtooth appearance and hyporeflective internal contents; and a type 3 pattern, showing broad, mound-shaped elevations of the RPE-basal laminar band with hyporeflective internal contents^4^.

The presence of large drusen, drusenoid PED, and macular complications, including GA, CNV, and AVL, was also assessed^4,19^. Large drusen was defined as at least 3 times the greatest height or diameter of typical cuticular drusen^4^. Drusenoid PED was defined as one-half disc diameter of confluent soft drusen under the center of the macula^25^. GA was defined as unifocal or multifocal sharply demarcated hypopigmented areas with large choroidal vessels visible due to RPE atrophy, observed on CFP images and hypoautofluorescence with a diameter of at least 175 μm observed on FAF images^26–28^. CNV was considered to be present if subretinal hemorrhage or fluid with subretinal tissue was observed on CFP and OCT images. A disciform scar was also included as evidence of old CNV. By using OCT, FA, and ICGA, CNV was classified into types 1, 2, and 3 using the Gass–Freund classification^1,29^. AVL was considered to be present if a yellowish subretinal material was observed in CFP images and hyperautofluorescent collections within the macular area, not secondary to vitelliform macular dystrophy, were observed on FAF images. These corresponded to hyperreflective lesions between the RPE-Bruch’s membrane band and the external limiting membrane seen on OCT images^30,31^.

Peripheral cuticular drusen are reported to be associated with senile reticular pigmentary degeneration^10,12^. Peripheral reticular pigmentary degeneration was defined as a reticular pattern of degenerated lesions that appeared hypofluorescent in the peripheral fundus areas on FA images^32,33^. The presence of peripheral pigmentary degeneration was evaluated and its prevalence according to the distribution pattern was compared.

Imaging features of cuticular drusen, including involvement of the peripapillary region and the total retinal area occupied by cuticular drusen exceeding 5 disc-areas, were also analyzed^4^. To investigate the relationship between aging and the prevalence of macular complications, patients were divided into age groups ≤ 60 years and > 60 years. The prevalence of macular complications was statistically compared between these two groups.

Morphological and topographic characteristics of cuticular drusen were assessed by two investigators (D.H.S. and M.K.), and in case of disagreement, a senior interpreter (D.I.H.) made the final decision. Statistical analyses were performed with SPSS software version 25.0 (SPSS, Inc, Chicago, IL, USA). A P-value less than 0.05 was considered statistically significant.

### Ethics approval and patient consent

This study was performed in accordance with the principles of the Declaration of Helsinki. It was approved by the institutional review board at Samsung Medical Center. Given the retrospective nature of the study and the use of anonymized data, requirements for informed consent were waived by the institutional review board.

## Data Availability

The datasets generated during and/or analyzed during the current study are available from the corresponding author on reasonable request.
